# A Unique Expression Profile Responding to Powdery Mildew in Wild Emmer Wheat D430

**DOI:** 10.3390/ijms26010242

**Published:** 2024-12-30

**Authors:** Yintao Dai, Ningning Yu, Hongxing Xu, Shaoqing Liu, Jiadong Zhang, Ruishan Liu, Jiatong Li, Yaoxue Li, Bei Xiao, Guantong Pan, Dongming Li, Cheng Liu, Yuli Jin, Pengtao Ma

**Affiliations:** 1 Yantai Key Laboratory of Characteristic Agricultural Biological Resources Conservation and Germplasm Innovative Utilization, College of Life Sciences, Yantai University, Yantai 264005, China; 2 School of Life Sciences, Henan University, Kaifeng 475004, China; 3 Yantai Academy of Agricultural Sciences, Yantai 265500, China; 4 National Key Laboratory of Wheat Breeding, Key Laboratory of Wheat Biology and Genetic Improvement in the North Huang-Huai River Valley, Crop Research Institute, Shandong Academy of Agricultural Sciences, National Engineering Research Center for Wheat and Maize, Shandong Wheat Technology Innovation Center, Jinan 250100, China; lch6688407@163.com; 5 National Center of Technology Innovation for Comprehensive Utilization of Saline-Alkali Land, Dongying 257347, China

**Keywords:** wild emmer wheat, powdery mildew, gene expression, mapping, BSR-Seq analysis

## Abstract

Powdery mildew, caused by *Blumeria graminis* f. sp. *tritici* (*Bgt*), is a disease that seriously harms wheat production and occurs in all wheat-producing areas around the world. Exploring *Pm* gene(s) and developing resistant cultivars are preferred to control the disease. Wild emmer wheat (*Triticum dicoccoides*, 2n = 4x = 28, AABB) has accumulated abundant gene resources for resistance to powdery mildew during the long process of natural evolution. In the current study, the WEW accession D430 was highly resistant to powdery mildew at the whole-growth stage. Genetic analysis showed that the powdery mildew resistance in D430 was conferred by a single dominant locus or gene by the cross of D430 and susceptible durum wheat 647, tentatively named *PmD430*. Combining BSR-Seq analysis, molecular mapping, and sequence alignment, *PmD430* was finally mapped to *Pm4* locus, and the sequence was identical to *Pm4b*. Subsequently, 1871 DEGs between resistant and susceptible bulks were annotated and analyzed by Gene Ontology (GO) and Kyoto Encyclopedia of Genes and Genomes (KEGG) pathway enrichment. Eight disease-related genes were evaluated by qRT-PCR and exhibited a unique expression pattern when invaded by *Bgt* isolate E09 and was, therefore, presented as latent targets for regulating powdery mildew resistance in D430.

## 1. Introduction

Powdery mildew, caused by *Blumeria graminis* f. sp. *tritici* (*Bgt*), is a disease that seriously harms wheat production and occurs in all wheat-producing areas around the world [1]. It is most severe in the United States and Western Europe; in China, it has also spread to the wheat regions in the middle and lower reaches of the Yangtze River, the Huang Huai Hai region, and other major wheat-producing areas. In 1981, there was a large-scale national outbreak of wheat powdery mildew, with an affected area of nearly three million hectares [2]. In the affected wheat fields, the yield generally decreased by 5–10%, and in severe cases, the yield decreased by 30–50% [3]. Therefore, there is an ongoing effort in identifying new sources of resistance for breeding programs.

To date, more than 140 formally designated and provisionally named *Pm* genes were discerned from common wheat and other species [4,5]. Among them, at least a third of the reported *Pm* genes were derived from wheat relatives, such as *Thinopyrum intermedium*, *Th. Ponticum*, *Dasypyrum villosum*, and *Aegilops* [5]. Up to now, only 26 *Pm* genes were cloned, including 19 genes encoding NLR immune receptors, 5 genes encoding kinase protein, and 2 genes encoding transporter [5]. As a closely related species of wheat, wild emmer wheat (*Triticum dicoccoides*, 2n = 4x = 28, AABB, abbreviated as WEW) has accumulated abundant gene resources for resistance to powdery mildew during the long process of natural evolution. *Pm* genes *Pm26* [6], *Pm42* [7], *Pm36* [8], *Pm41* [9], *Pm64* [10], *Pm69* [4], and *MlNFS10* [11] were first identified in WEW. Through the exploration of the *Pm* genes in WEW, we can gain an in-depth understanding of the molecular mechanism against powdery mildew and provide a theoretical basis for disease resistance breeding. At the same time, by using these gene resources, new wheat cultivars with high resistance to powdery mildew can be cultivated, further improving the yield and quality of wheat. In addition, it can also provide references for the disease resistant breeding of other crops.

Bulked segregant RNA sequencing (BSR-Seq) can analyze a large number of transcripts simultaneously to screen out the differentially expressed genes (DEGs) in disease-resistant and disease-susceptible wheat samples [12,13,14]. These DEGs may be directly involved in the disease-resistant reaction process of wheat. Performing functional annotation and enrichment analysis on the DEGs is helpful to reveal the molecular mechanism of wheat disease resistance. Ma et al. (2021) investigated the potential resistance components and identified six disease-related genes as key candidates for the dissection of powdery mildew resistance mechanisms in wheat accession YD588 using BSR-Seq analysis [15]. Zhu et al. (2020) mapped *PmJM23* at the *Pm2* locus on chromosomal arm 5DS and analyzed 3803 DEGs between Jimai 23 and susceptible parent Tainong 18 by Gene Ontology (GO), Clusters of Orthologous Groups (COG), and Kyoto Encyclopedia of Genes and Genomes (KEGG) pathway enrichment [16]. Qian et al. (2024) discovered that nine powdery mildew resistance-related genes were distinctly expressed after *Bgt* inoculation and were considered as key regulatory genes in durum wheat W762 [17]. In the study of fusarium head blight (FHB) resistance, Xu et al. (2020) found that some genes associated with the plant defense hormone signaling pathway were significantly up-regulated in disease-resistant parent Xinong 979 by RNA-Seq analysis, indicating that these signaling pathways may play a crucial role in the resistance process against FHB [18].

The WEW accession D430 was resistant to powdery mildew at the whole-growth stage. To understand the molecular mechanism against powdery mildew and provide a theoretical basis for disease resistance breeding, our study intended to (i) deliberate the inheritance law to powdery mildew, (ii) explore the candidate interval and key candidate gene(s) in D430, and (iii) profile the key regulatory genes mediating powdery mildew resistance by BSR-Seq analysis and qRT-PCR.

## 2. Results

### 2.1. Evaluation and Genetic Analysis of Powdery Mildew Resistance in D430

**Figure 1:**
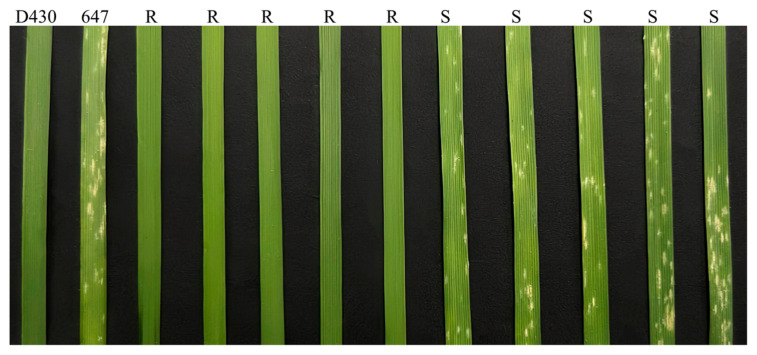
Responses of resistant parent D430, susceptible parent 647, and part of F_2:3_ plants at ~14 days post inoculation with *Bgt* isolate E09. R, resistant to powdery mildew; S, susceptible to powdery mildew.

When inoculated with *Bgt* isolate E09, WEW accession D430 showed no visible symptoms and was immune with infection types (IT) 0; in comparison, durum wheat accession 647 was extremely susceptible with IT 4 (Figure 1). All the 10 F_1_ plants of the cross D430 × 647 were resistant with ITs 0–1. For the 202 F_2_ plants, 153 were resistant, and 49 were susceptible, which fitted the Mendelian monogenic segregation theoretical ratio (χ^2^ = 0.02; *p* = 0.87). A total of 202 derivative F_2:3_ families further verified single gene inheritance with a segregation ratio of 47 homozygous resistant–106 segregating–49 homozygous susceptible families (χ^2^ = 0.53; *p* = 0.76) (Figure 1, Table 1). Accordingly, the resistance against *Bgt* isolate E09 in D430 was conferred by a single dominant gene, tentatively designated as *PmD430*.

### 2.2. BSR-Seq Analysis Revealed High-Quality Sequence Data

After BSR-Seq analysis, 21.46 Gb clean data for resistant bulk were obtained with the quality index of Q30 > 95% and GC content from 42 to 72%. Equally, 23.96 Gb clean data for susceptible bulk were obtained with the quality index of Q30 > 96% and GC content from 38 to 70%. Following sequence alignment, clean reads of 143,475,520 for R bulk and 160,241,474 for S bulk were mapped on the wild emmer v2.0 reference genome, respectively. And the high-quality sequence data were suitable for the subsequent analysis.

### 2.3. Confirmation of Candidate Interval in D430

**Figure 2:**
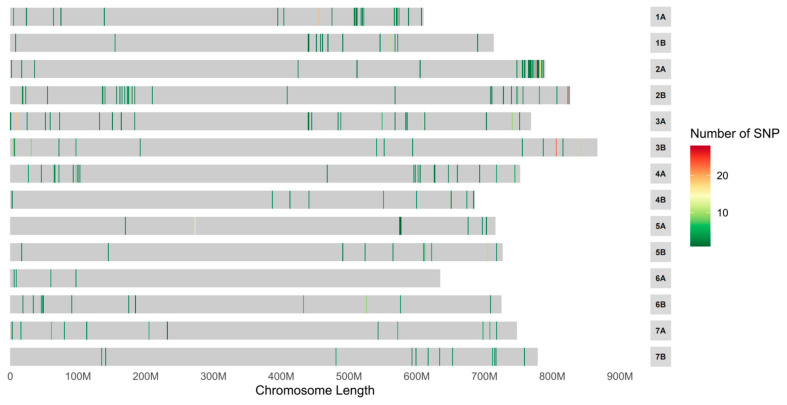
Distribution of polymorphic SNPs between resistant (R) and susceptible (S) pools on 14 wheat chromosomes.

**Figure 3:**
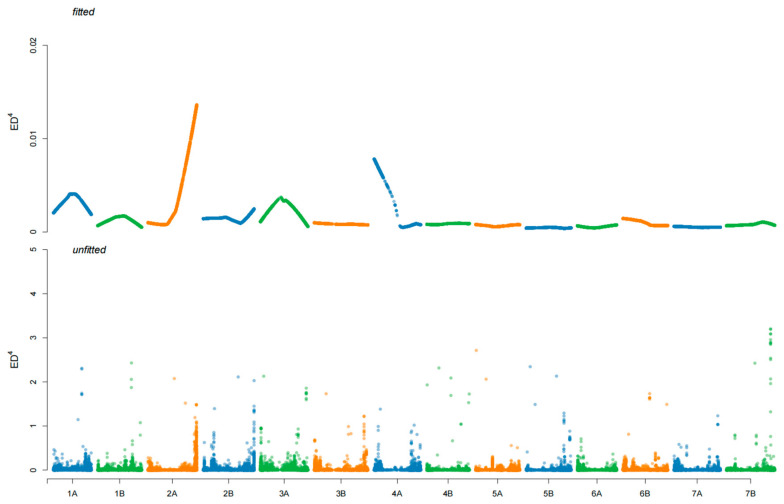
The ED map of 14 wheat chromosomes in candidate regions was analyzed.

A great deal of 3156 SNPs between the two bulks was detected with SNP index analysis. According to the Δ SNP index value, only one estimated candidate region was detected, located at the end of chromosome arm 2AL (Figure 2). To further ensure the result, the ED algorithm was also carried out, and the same results were obtained (Figure 3). A series of *Pm4* alleles were reported in this region [5]. To confirm this result, *JS717*/*JS718*, the diagnostic functional marker for *Pm4* locus, was used to genotype 202 F_2:3_ families of D430 × 647. The result showed that *JS717*/*JS718* was co-segregated with the *PmD430*, indicating that *PmD430* was likely to be an allele at *Pm4* locus. To further determine *PmD430*, the full-length genome sequence of *Pm4*-homologous sequence in accession D430 was amplified and sequenced. And the results showed that the homologous sequence in D430 was the same as *Pm4b*. In conclusion, the resistance against powdery mildew in D430 was most likely conferred by *Pm4b*, an allele at the *Pm4* locus.

### 2.4. Discovery and Classification of DEGs

**Figure 4:**
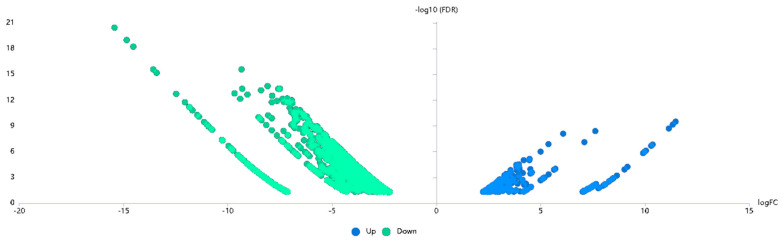
Distribution of the SNPs on 14 chromosomes based on the Euclidean distance (ED) algorithm.

However, it did not completely rule out that there may be other resistance-related gene(s) closely linked to *Pm4b* at the *PmD430* locus. In the candidate interval of *PmD430*, more than 291 high-confidence SNPs between resistant and susceptible bulks were present. These SNPs were employed for further DEG analysis. Based on BSR-Seq analysis, a total of 1871 DEGs were detected between two bulks, of which 1598 DEGs were down-regulated and 273 DEGs were up-regulated using the expression index of susceptible bulk as a standard (Figure 4). Upon further analysis, only 675 DEGs were located in this candidate interval of 755.5–783.6 Mb. These genes are considered key candidate genes in the resistance response to powdery mildew in D430.

### 2.5. DEG Enrichment Analyses of GO and KEGG Pathway

**Figure 5:**
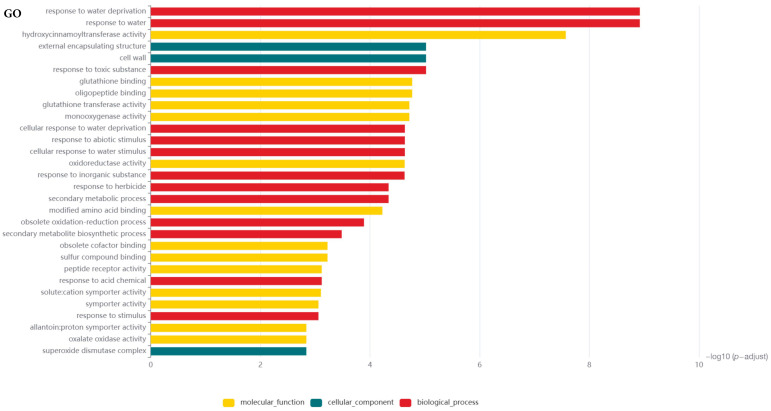
Gene Ontology (GO) analysis of the differentially expressed genes (DEGs).

**Figure 6:**
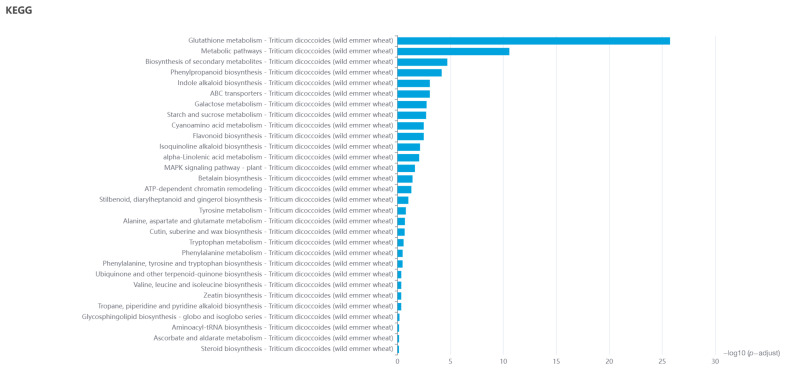
Kyoto Encyclopedia of Genes and Genomes (KEGG) pathway analysis for differentially expressed genes (DEGs).

The current GO analysis provides a powerful tool for understanding biological reaction processes, cellular components, and molecular functions. Initially, DEGs were analyzed through differential expression analysis. Research shows that these DEGs principally involve biological activities such as water scarcity adaptation, response to abiotic stress, secondary metabolic pathways, and reply to external stimuli; cell components, including external encapsulating structure and superoxide dismutase complex; molecular function, including hydroxycinnamoyltransferase activity, glutathione binding, and monooxygenase activity. Notably, there was DEG enrichment in the biological progress, indicating these genes may play a key role in the disease defense process (Figure 5). In order to further explore metabolic pathway information and understand the molecular mechanisms of biological processes that may involve DEGs, we conducted KEGG pathway enrichment analysis on DEGs in differential expression analysis. A total of 332 significantly enriched pathways inferring 30 categories in metabolic pathway, environmental processing, cellular processes, and genetic information processing were found (Figure 6).

### 2.6. Verification of Disease-Resistance-Related Genes by qRT-PCR in D430

**Figure 7:**
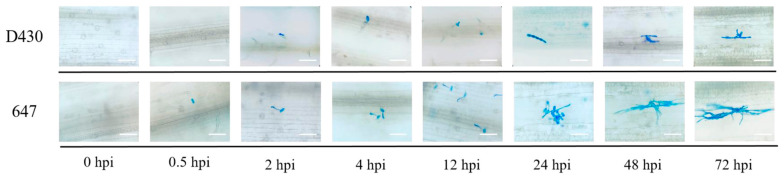
Infection process of *Blumeria graminis* f. sp. *tritici* (*Bgt*) isolate E09 on leaves of D430 and 647. Wheat leaf samples were taken at different times post inoculation (hpi) for Coomassie blue staining. Scale bar, 200 μm.

**Figure 8:**
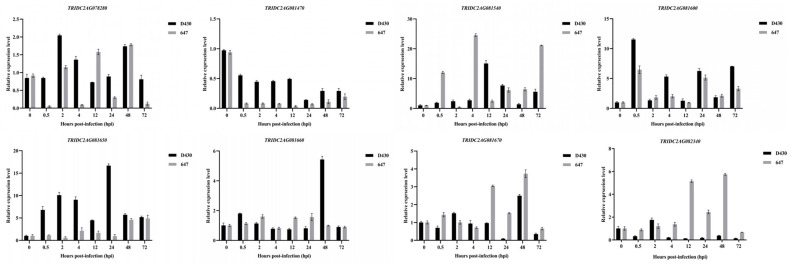
Expression patterns of *TRIDC2AG078280*, *TRIDC2AG081470*, *TRIDC2AG081540*, *TRIDC2AG081600*, *TRIDC2AG081650*, *TRIDC2AG081660*, *TRIDC2AG081670*, and *TRIDC2AG082340* in D430 and 647 at 0, 0.5, 2, 4, 12, 24, 48, and 72 h post inoculation with *Bgt* isolate E09.

To further determine key genes during the process of resistance response to powdery mildew in accession D430, we first surveyed the invasion process with the *Bgt* isolate E09. As shown in Figure 7, 0 h post inoculation (hpi) was considered blank control, the time period from 0.5 to 12 hpi as the stage of formation and penetration of primary germ tube, 24 hpi as the formation stage of haustorium, 48 hpi as the stage of secondary penetration, and 72 hpi as the stage of microcolony formation (Figure 7). Based on the results, we profiled the expression pattern of eight DEGs located in this candidate interval of 755.5–783.6 Mb at different hpi with *Bgt* isolate E09. Eight of the candidate genes presented significant differences between the resistant D430 and the susceptible accession 647 when inoculating *Bgt* isolate E09. The transcription levels of four genes *TRIDC2AG078280* (encoding Beta-fructofuranosidase), *TRIDC2AG081600* (encoding disease resistance protein), *TRIDC2AG081650* (encoding disease resistance protein), and *TRIDC2AG081660* (encoding disease resistance protein RGA2) were up-regulated in D430 but not in 647 within 0–72 hpi. However, three genes *TRIDC2AG081540* (encoding disease resistance protein), *TRIDC2AG081670* (encoding disease resistance protein), and *TRIDC2AG082340* (encoding disease resistance protein RGA2) were up-regulated in susceptible parent 647 but not in D430 with *Bgt* inoculation. Notably, the expression level of gene *TRIDC2AG081470* (encoding disease resistance protein) was down-regulated within 0–72 hpi (Figure 8).

## 3. Discussion

As the ancestral species of cultivated tetraploid and hexaploid wheat, WEW serves as a valuable source, providing genes for disease resistance, adaptability, and quality. Numerous resistance genes were reported in WEW accessions, such as leaf rust resistance genes *Lr53* [19] and *Lr64* [20]; stripe rust resistance genes *Yr15* [21], *Yr35* [22], *Yr36* [23], and *YrH52* [24]; and *Pm* genes *Pm26*, *Pm42*, *Pm36*, *Pm41*, *Pm64*, *Pm69*, and *MlNFS10* [4,6,7,8,9,10,11]. Introducing these genes into cultivated wheat play an important role in wheat genetic improvement. WEW accession D430 showed high resistance to powdery mildew at the whole-growth stage. In the present study, we identified a dominant *Pm* gene *PmD430* at the *Pm4* locus on chromosome arm 2AL in D430, revealing a potentially valuable genetic resource for breeding.

Genetic analysis indicated that the resistance to powdery mildew *Bgt* isolate E09 in D430 was conferred by a single dominant gene or locus. However, two candidate intervals 755.5–783.6 Mb on chromosome arm 2AL and 0–13.4 Mb on the chromosome arm 4AS were preliminarily identified by using BSR-Seq analysis. Then, using molecular markers from BSR-Seq, *PmD430* was finally mapped to a 1.2 cM genetic interval corresponding to 762–768 Mb physical interval on chromosome arm 2AL based on the reference genome of wild emmer v2.0, in which *Pm4* was identified. For the *Pm4* locus, eight alleles *Pm4a*–*4h* were reported [25]. Among them, *Pm4c* (*Pm4c* = *Pm23*), *Pm4d*, *Pm4e*, *Pm4f*, *Pm4g*, and *Pm4h* were derived from hexaploid wheat accessions, and *Pm4a* and *Pm4b* were reported in tetraploid wheat (T. *turgidum*) cultivars and tetraploid T. *carthlicum*, respectively [25]. *Pm4b*, encoding chimeric kinase-multiple C2 domains and transmembrane region protein, was first cloned using the strategy of MutChromSeq. A haplotype diagnostic marker (*JS717/JS718*) was developed and used to target stack *Pm4* alleles [25]. In our study, the sequence of *PmD430* was found to be identical to *Pm4b* using homology-based cloning. Additionally, the diagnostic marker *JS717/JS718* of *Pm4* locus was co-segregated with *PmD430* in the 202 F_2:3_ families of cross D430 × 647. Therefore, *PmD430* was likely *Pm4b*. However, the current mapping results using the population of 202 F_2:3_ families of cross D430 × 647 did not completely rule out the possibility of other resistance-related gene(s) tightly linked to *Pm4b* at the *PmD430* locus. The unique inheritance of *PmD430* might be governed by a particular resistance mechanism that has the potential to augment the diversity in resistance types. It can also provide a valuable new resource for disclosing the expression of multiple genes or alleles within or around the same locus. In the future, the analysis of the mapping interval and the interaction relationship among related genes will be beneficial in elucidating this complex issue. More genetic investigations and various cross with different susceptible parents are required to enhance the current comprehension of the mechanism and utilize *PmD430* in wheat breeding. Once applied to the breeding process, *PmD430* presents the advantage of more straightforward selection compared to the situation with recessive genes.

Plant resistance represents a complex procedure during the interaction between the host and the pathogen [26]. To perceive the invasion of pathogen and activate various defense responses, numerous genes at various layers will be activated within host plants, including the cell wall, the plasma membrane, and diverse enzymes in the cytoplasm [27,28,29]. In the present study, when the *Bgt* isolate invasion, a large number of DEGs were appraised and investigated use GO analysis and KEGG enrichment analysis. Among them, three categories of genes, concerned with cellar component, biological process, and molecular function, were enriched with a high proportion. This result conformed to the mechanisms model of signal transduction and defense activation: once pathogens intrude, signal transduction pathways are activated; then, defense mechanisms are mobilized to combat the *Bgt* invasion; all these processes need energy provided by metabolism and biosynthesis.

## 4. Materials and Methods

### 4.1. Plant Materials

The WEW accession D430 was resistant to powdery mildew, while the durum wheat accession 647 which was susceptible to all the *Bgt* isolates, was employed as the susceptible parent to make a cross with D430, resulting in the generation of F_1_, F_2_, and F_2:3_ progeny for genetic analysis and BSR-Seq analysis. They were sourced from Professor Hongxing Xu, School of Life Sciences, Henan University, Kaifeng, China, and preserved on our lab. This study selected the wheat cultivar Tainong 18, which is sensitive to multiple *Bgt* isolates, as the sensitive control cultivar for phenotype evaluation experiments.

### 4.2. Genetic Analysis

The population F_1_, F_2_, and F_2:3_ progenies of the cross D430 × 647, along with resistant parent D430 and susceptible parent 647, were inoculated with *Bgt* isolate E09 for genetic analysis. Moreover, 72-cell rectangular trays were used to plant seedling samples, and 5–8 seeds were sown in each cell. For the F_2:3_ families, at least 20 seeds per F_2:3_ family were evaluated. The susceptible check, Tainong 18, was sown randomly in each tray. The conditions for the incubator are set as follows: 20 °C/14 h/day and 18 °C/10 h/night. Once the seedlings had grown to the two-leaf stage, they were inoculated with fresh conidiospores by using sweeping method, immediately incubated in a dark environment and 100% humidity at 18 °C for 24 h. Next, the conditions for the incubator are set as follows: 20 °C/14 h/day and 18 °C/10 h/night. About 10 days later, when the initial leaf of the susceptible control cultivar, Tainong 18, was entirely coated with spores, ITs were scored using the 0–4 scale described by Jin et al. (2022) [30]. Among them, ITs 0, 0, 1, and 2 are classified as resistant phenotypes, while ITs 3 and 4 are considered as susceptible phenotypes [30,31]. The phenotypic assessment was repeated thrice using the same procedure to guarantee the fidelity of data.

### 4.3. RNA Extraction and Sequencing Library Preparation for BSR-Seq

After phenotypic assessment, 30 homozygous resistant and homozygous susceptible individuals were randomly selected to construct resistant RNA bulk and susceptible RNA bulk, respectively. Total RNA was extracted from the young leaf tissues using the RNAsimple Total RNA Kit (Tiangen, Beijing, China) following the operating instructions. Resistant and susceptible RNA pools were formed by mingling equivalent amounts of RNA from 30 homozygous resistant plants and 30 homozygous susceptible plants of F_2:3_ families separately. RNA quality is assessed using methods such as agarose gel electrophoresis and spectrophotometry. Next, mRNA is purified from the total RNA using oligo (dT) beads. The purified mRNA is then fragmented by chemical or enzymatic methods. After fragmentation, cDNA is synthesized using reverse transcriptase and random primers. The cDNA is then end-repaired and ligated to adapters. The ligated products are amplified by PCR to generate the final sequencing library. The library is quantified and quality checked to ensure it meets the requirements for sequencing.

### 4.4. Sequencing and Data Analysis

Sequencing was performed on the Illumina HiSeq sequencing platform (Illumina HiSeq4000) at Tcuni Bioscience (Chengdu, China). Large amounts of sequencing data were generated in the form of short reads. The raw data were filtered by checking read quality scores, removing low-quality reads, and trimming adapter sequences. Then, the clean data were aligned to the reference genome of wild emmer v2.0 using the software STAR for subsequent SNP calling, gene annotation, differential gene expression analysis, and functional enrichment analysis [32].

### 4.5. Gene Mapping

SNP calling was performed using the tools GATK v4.2.3.0 and SAMtools v1.17 [33]. The allele frequency of each SNP which refers to the occurrence frequency of a certain allele in a population was calculated in the resistant pool and the susceptible pool, respectively. Based on the difference in allele frequencies, SNP with significant differences between the resistant pool and the susceptible pool may be closely linked to the target gene in D430. Then, the linkage relationship between these differential SNPs and target gene(s) was investigated using methods such as Bayesian analysis and others. Finally, these candidate genes within the determined linkage interval were considered as the key genes related to resistance against powdery mildew.

### 4.6. Gene Cloning and Sequence Alignment

Considering that the powdery mildew resistance gene in D430 is limited to the *Pm4* locus, the functional marker *JS717/JS718* of *Pm4* were used to genotype 277 F_2:3_ families of D430 × 647 to decide the relationship between *Pm4* and powdery mildew resistance genes in D430. Furthermore, the homologous sequence in D430 was also amplified using the primers JS256, JS257, JS251, JS278, JS261, and GH407 following the protocol described by Sánchez-Martín et al. (2021) [25] and subsequently aligned with the sequences of *Pm4a*–*4e* using the software TBtools v1.045 [34].

### 4.7. Differential Expression Gene Analysis, Functional Annotation, and Enrichment Analysis of the DEGs

The number of reads mapped to each gene or transcript were counted to quantify the expression levels using software such as HTSeq v2.0.3 or featureCounts v2.0.1. The expression levels between the resistant and susceptible pools were compared by employing statistical methods, and DEGs were detected by the EBSeq v3.5 software with the following standard: fold change ≥ 2 and FDR (false discovery rate) < 0.01. The identified DEGs were annotated to determine their functions and biological pathways using databases such as GO, KEGG, and others for annotation with an R package for DEGs [35]. The enrichment analysis of the DEGs was performed in specific biological processes, cellular components, or molecular functions.

### 4.8. Sample Preparation and qRT-PCR

qRT-PCR was carried out to identify the expression level of the DEGs related to the powdery mildew resistance in D430. Following inoculation of D430 and 647 with *Bgt* isolate E09, samples of the leaves were collected at 0, 0.5, 2, 4, 12, 24, 48, and 72 hpi with three parallel experiments. Total RNA was extracted using RNAsimple Total RNA Kit (Tiangen, Beijing, China). Around 2 μg of RNA was reverse transcribed to cDNA with a FastQuant RT Kit (Tiangen, Beijing, China). qRT-PCR assays were performed using SYBR Premix Ex Taq (Takara, China) using the instrument Bio-Rad CFX Connect real-time PCR system (BIO-RAD, Hercules, USA). The specific primers for target genes were designed according to the coding sequences and developed using Primer 5 software. Relative gene expression was calculated by the 2^−∆∆Ct^ method [36]. *TaActin* gene was used as the internal control for normalization. Three technical replications were performed for each sample.

To further monitor the invasion process, D430 and 647 were inoculated, and *Bgt* isolate E09 and 2 cm seedling leaves of post-*Bgt* infection at 0, 0.5, 2, 4, 12, 24, 48, and 72 hpi were sampled and fixed in 5 mL of Carnoy’s fixative (absolute alcohol–glacial acetic acid, 3:1, *v*/*v*) at 37 °C for 24 h and stained in 5 mL of 0.6% (*w*/*v*) Coomassie blue liquor for 5 min. Excess dye on the leaf samples was carefully rinsed with distilled water [36]. Finally, the specimens were inspected under an Axioscope 5 microscope (ZEISS, Oberkochen, Germany).

## 5. Conclusions

Wild emmer wheat accession D430 exhibits strong resistance to powdery mildew throughout its entire growth stage. In the current study, we elucidated the resistance genetic pattern to powdery mildew, identified DEGs by BSR-Seq analysis, and characterized the expression level of eight key genes related to resistance against powdery mildew.

## Figures and Tables

**Table 1:** Segregation ratios of F_2_ and F_2:3_ generations of D430 and 647 following inoculation with *Blumeria graminis* f. sp. *tritici* (*Bgt*) isolate E09 at the seedling stage.

Parent and Cross	Generation	Observed Ratio	Expected Ratio	*χ* ^2^	*p*
D430	R_P_	R:S = 10:0			
647	S_P_	R:S = 0:10			
D430 × 647 F_1_	F_1_	R:S = 10:0			
D430 × 647 F_2_	F_2_	R:S = 153:49	3:1	0.02	0.87
D430 × 647 F_2:3_	F_2:3_	HR:Seg:HS = 47:106:49	1:2:1	0.53	0.76

R_P_, resistant parent; S_P_, susceptible parent; R, resistant; S, susceptible; HR, homozygous resistant; Seg, segregating; HS, homozygous susceptible.

## Data Availability

The necessary data can be requested from the correspondence author.
